# 

**Published:** 1894-08-18

**Authors:** 


CONTENTS.

Lord Salisbury's Address - 403

Continental Notes from a Holiday Correspondent - 404

Bacteriology in the Diagnosis of Diphtheria - 405

The Educational Treatment of Idiots, Imbeciles, and Feeble-minded Children. By G. E. Shuttleworth, B.A., M.D. - 406

Annotations.—Radcliffe Infirmary, Oxford—The Medical Confessional—The Lungs of London - 407

Notes and News - 408

Medical Progress and Hospital Clinics:

On Malignant Tumours of the Rectum - 409

Periscope of Dermatology - 410

Progress in Surgery.—Genito-Urinary Surgery - 412

The British Medical Association at Bristol - 413

Hygiene in University Education. By John S. Billings, M.D., D.C.L., Oxon - 415

EXTRA SUPPLEMENT.—THE NURSING MIRROR.

News from the Nursing World.—Our Pauper Children—Unauthorised "Inspection"—Unsigned Accusations—Without Board—What Becomes of the Linen?—Nurses—Respiration versus Nourishment—Newton Abbot Workhouse—Queen's Nurse at Selkirk—Valuable Auxiliaries—Carrickmacross—Genuine Nurses—Short Items - cci

On General Nursing.—XXIV. Typhoid Fever (*continued*) cciii

Structure and Care of the Teeth.—II. - cciv

A Case for Assistance - cciv

Where to Go - cciv

Our American Letter - ccv

Queen Victoria's Jubilee Institute for Nurses - ccv

Notes from Germany - ccv

Notes from India - ccv

Holidays in Japan - ccvii

English Nurses on the Labrador - ccviii

Women Architects - ccviii

A Winter in the Sunny South.—Caithness - ccix

Everybody's Opinion - ccx

For Reading to the Sick - ccx

